# Short-Term Visual Deprivation, Tactile Acuity, and Haptic Solid Shape Discrimination

**DOI:** 10.1371/journal.pone.0112828

**Published:** 2014-11-14

**Authors:** Charles E. Crabtree, J. Farley Norman

**Affiliations:** Department of Psychological Sciences, Ogden College of Science and Engineering, Western Kentucky University, Bowling Green, Kentucky, United States of America; Emory University, United States of America

## Abstract

Previous psychophysical studies have reported conflicting results concerning the effects of short-term visual deprivation upon tactile acuity. Some studies have found that 45 to 90 minutes of total light deprivation produce significant improvements in participants' tactile acuity as measured with a grating orientation discrimination task. In contrast, a single 2011 study found no such improvement while attempting to replicate these earlier findings. A primary goal of the current experiment was to resolve this discrepancy in the literature by evaluating the effects of a 90-minute period of total light deprivation upon tactile grating orientation discrimination. We also evaluated the potential effect of short-term deprivation upon haptic 3-D shape discrimination using a set of naturally-shaped solid objects. According to previous research, short-term deprivation enhances performance in a tactile 2-D shape discrimination task – perhaps a similar improvement also occurs for haptic 3-D shape discrimination. The results of the current investigation demonstrate that not only does short-term visual deprivation not enhance tactile acuity, it additionally has no effect upon haptic 3-D shape discrimination. While visual deprivation had no effect in our study, there was a significant effect of experience and learning for the grating orientation task – the participants' tactile acuity improved over time, independent of whether they had, or had not, experienced visual deprivation.

## Introduction

By the 1880's, it was well known from experiments performed by David Ferrier [1] and Hermann Munk [2] that large portions of the primate and mammalian cerebral cortex were specifically devoted to visual and auditory functions. By the 1990's, extensive neurophysiological research [3]–[5] had identified and localized an abundance of sensory cortical areas: in the macaque monkey, for example, there were at least 25 distinct cortical areas devoted to vision, at least 8 devoted to somatosensory functions, and at least 5 devoted to audition. The evidence for these functional subdivisions of cerebral cortex is compelling. At the same time, however, this division of functionality is somewhat of an oversimplification. More recent research [6]–[12] has proven that the cerebral cortex is plastic and that significant changes in neuronal responsiveness to sensory information occur with long-term visual deprivation (e.g., blindness). For example, Kahn and Krubitzer [6] surgically removed both eyes from opossum pups at 4 days of age (it is important to keep in mind that opossums are mammals, with a cerebral cortex that is similar to other mammals). When the enucleated opossums' cortical functioning was assessed at adulthood, neurons in what would have been their primary visual cortex responded either exclusively to tactile input, exclusively to auditory input, or to both tactile and auditory input. Similarly, Lewis, Saenz, and Fine [7] have demonstrated that neurons within the occipital cortex of early-blind humans respond to both tactile and auditory stimuli. The occipital response to non-visual stimulation was much greater for their blind participants and was substantially reduced in sighted participants. It is clear that long-term visual deprivation (e.g., blindness), at least, is associated with substantive changes in cortical functioning.

Many studies have now found that blindness is associated with enhancements in behavioral performance for non-visual sensory and perceptual tasks [13]–[22]. Our own laboratory [13], for example, has recently demonstrated that blindness is associated with enhancements in both tactile acuity and haptic solid shape discrimination. The cortical changes in functionality that accompany blindness probably contribute to such enhancements. Blindness-associated improvements in performance occur for tactile and haptic tasks [13]–[17], as well as for those involving audition [18]–[20] and olfaction [21], [22]. If long-term visual deprivation (months or years) can enhance sensory performance in non-visual modalities, what length of deprivation is required? The evidence acquired to date suggests that the duration of deprivation can be surprisingly short. Medium-length visual deprivation of 5 days to a week has been shown to produce enhancements in performance for tactile tasks [23]–[25]. Given that a period of 5 days is probably insufficient for significant changes in patterns of neuronal connectivity *per se*, Merabet et al. [24] suggested that the improvement obtained for their tactile task was likely caused by “an unmasking of latent multimodal connections underlying the recruitment of occipital cortex for tactile processing”. In this view, there are pre-existing tactile inputs to “visual” cortex in all of us, but in everyday life visual input dominates and “masks” the tactile input. When visual deprivation occurs (for example, by blindfolding for hours or days), the tactile input to visual cortex is “unmasked” – the “visual” cortex then becomes responsive to tactile input and contributes to the performance obtained for tactile behavioral tasks.

Evidence now exists to indicate that performance for some tactile tasks can be facilitated by only a few hours of visual deprivation. For example, Weisser et al. [26] found that participants who had been blindfolded for two hours exhibited superior performance (11.9 percent higher accuracy) relative to non-visually-deprived participants for a tactile task involving the discrimination of 2-D shape (i.e., discriminating between upside-down letters “T” and “V”). Whether similar improvements in tactile acuity can occur following short-term visual deprivation is not yet clear. Facchini and Aglioti [27] found that their participants' grating orientation discrimination thresholds decreased by about 21 percent after a 90-minute period of visual deprivation – this decrease did not occur for their non-deprived participants. Leon-Sarmiento et al. [28] similarly found grating orientation thresholds to decrease (by 23 percent) after 45 minutes of visual deprivation. Concern has been expressed [29] about the validity of the findings of both Facchini and Aglioti [27] and Leon-Sarmiento et al. [28]. Wong et al. [29], for example, pointed out that the “non-deprived” participants in the study by Facchini and Aglioti [27] were actually blindfolded and visually deprived whenever their tactile acuity was being measured. These researchers [29] argued that the performance of Facchini and Aglioti's “non-deprived” participants was not therefore necessarily representative of how they might have actually performed under non-deprived (i.e., sighted) conditions (which was never tested). Wong et al. [29] also pointed out that there was no control group in the study by Leon-Sarmiento et al. [28]. The tactile acuity of their visually deprived participants did improve after 45 minutes of blindfolding, but because there was no control group of non-deprived participants with which to compare, this improvement could have occurred simply as a result of practice and increasing experience with the task. In their own study (which did employ control groups of non-deprived participants, who were tested under sighted conditions), Wong et al. [29] found no improvement in grating orientation discrimination following up to 110 minutes of visual deprivation. In the current experiment, we sought to determine whether there is or is not an effect of short-term visual deprivation on tactile acuity. In addition, we evaluated the possible effect of short-term deprivation on the haptic discrimination of solid object shape. No study to date has simultaneously investigated the potential effects of short-term visual deprivation upon both tactile acuity and haptic shape discrimination.

## Materials and Methods

### Ethics Statement

The experiment was approved by the Western Kentucky University Human Subjects Review Board. The participants were students at Western Kentucky University, and all participants gave written consent prior to participation in the experiment.

### Participants

Twenty-eight younger adults participated in the experiment and were randomly divided into two groups. Fourteen participants (mean age was 21.2 years, SD  = 1.9) were visually deprived (i.e., blindfolded) throughout a large portion of the experiment, while 14 additional participants (mean age was 20.4, SD  = 0.6) were not visually deprived (i.e., possessed normal vision) and could see the entirety of our laboratory (approximately 400 ft^2^ containing furniture, tables, plants, etc). The blindfold eliminated all light when it was worn and was similar to those used in previous studies [24]–[27]. Our blindfold was especially similar to that used by Merabet et al. [24] and Kauffman et al. [25], in that our blindfold, while preventing all light from reaching the participants' eyes, also permitted normal eye and eyelid movement. As in the studies by Weisser et al. [26] and Wong et al. [29], our non-deprived participants were tested under sighted conditions.

### Apparatus

The order of presentation of the experimental stimuli was randomly determined for each participant by either an Apple iMac computer (for the tactile acuity task) or an Apple PowerMacintosh G4 computer (for the haptic solid shape discrimination task). The participants' judgments were entered into the computers for later analysis.

### Experimental Stimuli

**Figure 1:**
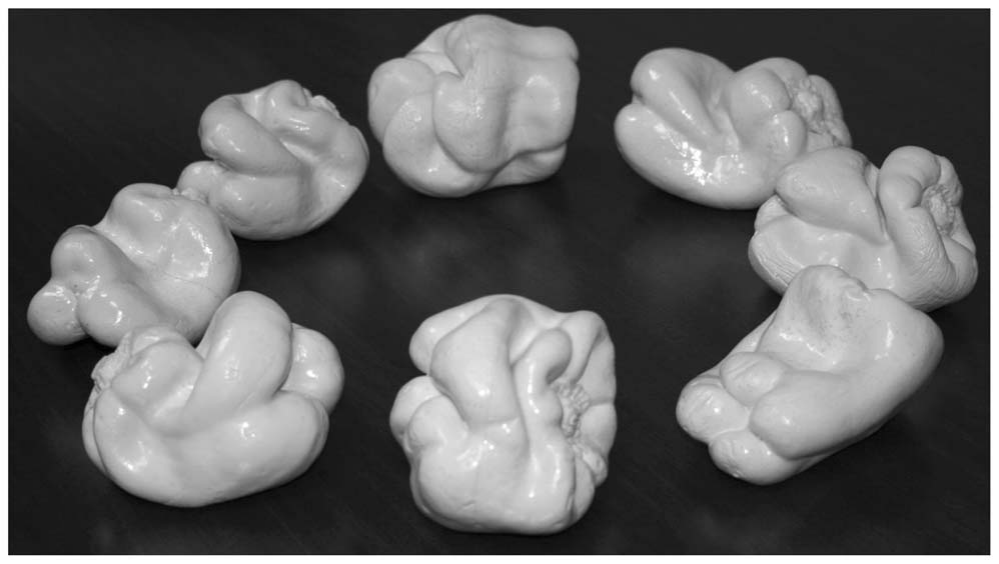
A photograph of the eight natural objects (bell peppers, *Capsicum annuum*) used as experimental stimuli for the solid shape discrimination task. Starting from the bottom left (going clockwise), the objects depicted are 1, 2, 3, 5, 7, 8, 11, and 12. These objects are a subset of those developed by Norman et al. [30].

The experimental stimuli for the tactile acuity task were tactile gratings (JVP Domes, Stoelting, Inc.), used previously by both our laboratory and others [13], [15], [24], [30]–[33]. The experimental stimuli used for the solid shape discrimination task were solid (plastic) copies of eight natural bell peppers, *Capsicum annuum*, that have been regularly used in previous investigations [13], [34]–[37]. Photographs of these stimulus objects are shown in Figure 1.

### Procedure

The tactile acuity of all participants was assessed at the beginning of the experiment. Following this, half of the participants were randomly assigned to the visual deprivation condition; these participants were then blindfolded (they remained blindfolded throughout the entire remainder of the experiment). After a 90-minute delay (during which the first author remained with all participants, deprived and non-deprived, and ensured that all participants remained alert by engaging in conversation and/or listening to background music), all participants' tactile acuity was measured again. Following the second evaluation of tactile acuity, the participants' ability to discriminate solid shape was assessed. After completing the second evaluation of their tactile acuity and the shape discrimination task, the visually deprived participants were finally (after about 2.5 to 3 hours) allowed to remove their blindfold. No participant in the visually deprived condition ever reported detecting any light whatsoever. Nevertheless, we felt that it was important to demonstrate and ensure the integrity of the blindfold. Throughout the experiment (e.g., immediately after putting on the blindfold, 30 minutes after the initial assessment of tactile acuity, & 85 minutes after the initial assessment of tactile acuity) we repeatedly tested each participant to ensure total light deprivation. The room light (a 75-watt incandescent lamp) was turned on and off according to a random schedule for at least ten trials on each occasion; the participants were asked to judge on each trial whether the room lights were on or off. The participants' detection performance was no better than chance at any of the three time periods (49.76, 43.57, & 48.57 percent correct light detection, chance  = 50.0 percent correct), demonstrating that the blindfold was effective in eliminating light.

The procedures used for determining the participants' grating orientation thresholds (i.e., their tactile acuity) were similar to those used both by ourselves [13], [30], [31] and others [24], [32], [33], [38]. On each trial, tactile gratings were manually applied to the distal fingerpad of the index finger for one second so that the grooves and ridges of the gratings were oriented either parallel or perpendicular to the long axis of the finger. The participants were required to judge whether each grating application was parallel or perpendicular. An occluding curtain prevented the sighted participants from seeing the application of the gratings to their fingertip (the curtain was present, however, when both the sighted and blindfolded participants were tested). The participants' ability to discriminate grating orientation was assessed in multiple blocks of 40 trials (twice the trials per block used by Van Boven and Johnson [38]). The participants' performance at the end of each block was expressed in terms of d′, which is the measure of perceptual sensitivity used in Signal Detection Theory [39]. D′ values were calculated from hit- and false-alarm rates [39]. A hit occurred when a grating was applied parallel to the fingertip and a participant correctly responded “parallel”; a false-alarm occurred when a grating was applied perpendicular to the fingertip, but a participant incorrectly responded “parallel”. Hit rates were determined [39] for each block by dividing the number of hits by the total number of parallel stimuli presented, while false-alarm rates were similarly determined [39] by dividing the number of false alarms by the total number of perpendicular stimuli presented. The participants were initially assessed using a groove width of 2 mm; as long as their orientation discrimination performance was above threshold (d′ = 1.35), subsequent blocks of 40 trials were run with smaller and smaller groove widths (e.g., 1.5, 1.2, 1.0, 0.75, & 0.5 mm). Once each participant's orientation discrimination performance dropped below a d′ of 1.35, linear interpolation was used [38] to determine their exact threshold (e.g., if a grating with a groove width of 1.0 mm produced a d′ value of 1.5 and a grating with a groove width of 0.75 mm produced a d′ value of 1.2, then that participant's threshold would be 0.875 mm).

The procedures used for the solid shape discrimination task were similar to those used by Norman et al. [13], [35]; as in these previous studies, the current experiment utilized a subset of the naturally-shaped stimuli developed by Norman, Norman, Clayton, Lianekhammy, and Zielke [37]. On every trial in the current experiment, the participants haptically explored two objects (behind an occluding curtain) for three seconds each, separated by a three second inter-stimulus-interval. The two objects presented on each trial either possessed the *same* shape or had *different* shapes. The participants were required to judge whether each pair of objects were the *same* or *different*. Only the most difficult pairs of objects to distinguish from our previous investigations were used: Objects 1 and 3, Objects 1 and 7, Objects 2 and 11, Objects 3 and 7, Objects 3 and 8, and Objects 5 and 12. Each participant judged a total of 96 pairs of objects (4 successive blocks of 24 trials). Within each block, half of the trials were devoted to the *different* object pairs, while the *same* object pairs were presented on the remaining trials (on a same trial, one of the 8 individual objects would be paired with itself). The order of same versus different trials was randomly determined within a block; however, each different pair was presented twice within a block. For each different pair, the order of presentation was randomized (e.g., for trials involving objects 3 & 7, object 3 would sometimes be presented first, while on other trials, object 7 would be presented first).

## Results

**Figure 2:**
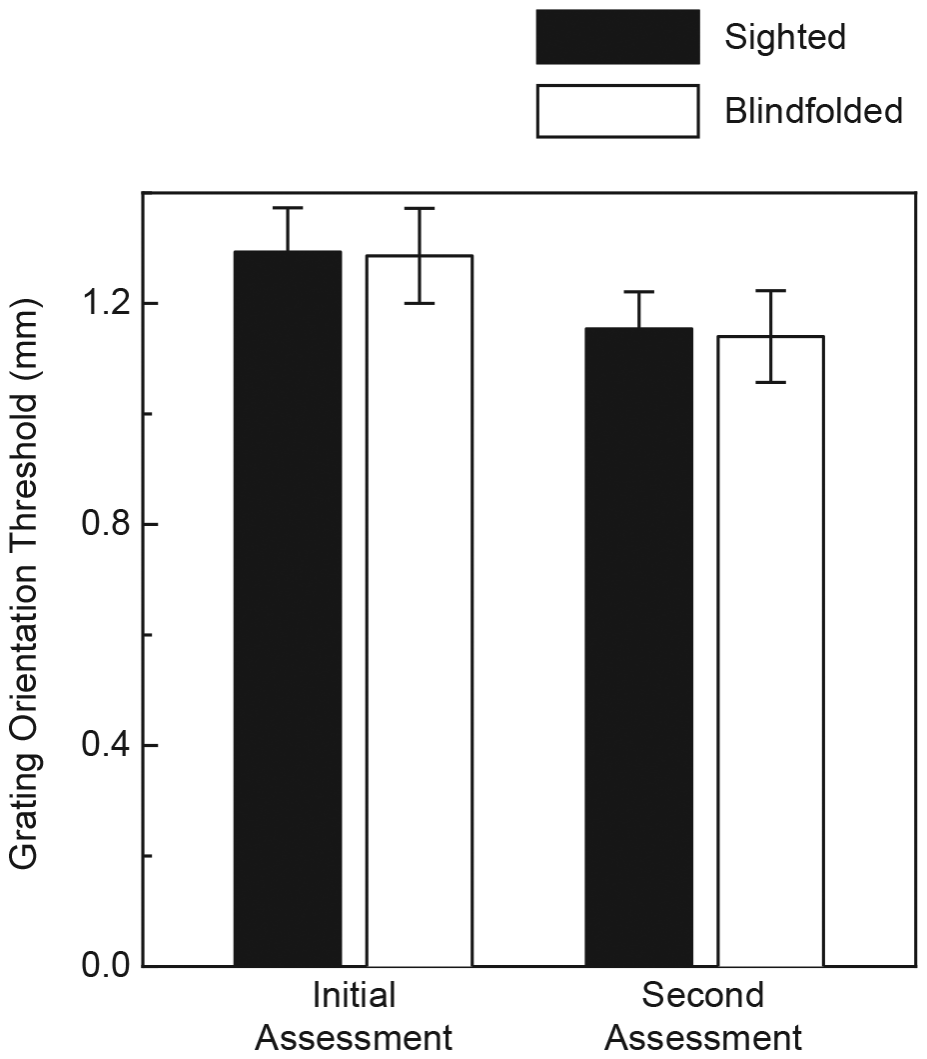
Experimental results. The participants' tactile acuity (grating orientation thresholds) measured both before and following a 90-minute period. Half of the participants were visually deprived of light (i.e., were blindfolded) during this 90-minute period. The filled bars indicate results obtained for the sighted participants, while the open bars indicate results obtained for the blindfolded (i.e., visually deprived) participants. The error bars indicate ±1 SE.

The participants' tactile acuities are plotted in Figure 2. According to the Shapiro-Wilk test for normality [40], the sighted and blindfolded participants' grating orientation thresholds were distributed normally for both the initial and second assessment of tactile acuity (i.e., the participants' thresholds did not deviate from normality: sighted, initial assessment, W(14)  = 0.94, p>.05; sighted, second assessment, W(14)  = 0.92, p>.05; blindfolded, initial assessment, W(14)  = 0.96, p>.05; blindfolded, second assessment, W(14)  = 0.93, p>.05). As can be readily seen from an inspection of Figure 2, there was a significant decrease in the participants' thresholds from the initial assessment to the second assessment (F(1, 26)  = 12.9, p = .001, partial 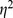 = .33). This improvement in performance for grating orientation discrimination is similar to that obtained for a different tactile task (grating groove width and ridge width discrimination) by Sathian and Zangaladze [41]. In their study, the participants' improvement in performance occurred for a dynamic tactile task (participants actively scanned grating surfaces), while our participants' improvement occurred for static touch. As Figure 2 clearly shows, there was a significant improvement in tactile acuity (i.e., lowered thresholds during the second assessment) for the blindfolded participants (open bars); however, there was an equal improvement in tactile acuity for the sighted control participants (filled bars). This equivalence in performance change for both groups of participants is reflected by a non-significant interaction between group and measurement session (F(1, 26)  = 0.007, p = .93, partial 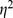 = 0.0). When considering this result, it is important to keep in mind that there was sufficient power to detect an effect of the visual deprivation had one existed: we had, for example, just as many sighted and visually-deprived participants as in the study by Facchini and Aglioti [27]. Furthermore, given the means and inter-participant variability shown in Figure 2, a power analysis [42] revealed that to have a 90 percent chance of detecting an effect this small (i.e., a difference in tactile acuity between the sighted and visually-deprived participants in the second assessment), we would need 8450 participants per group (16,900 total participants). It is clear that even if the extremely small difference in tactile acuity we observed between the sighted and deprived participants had been statistically significant (following a 90 minute period of light deprivation for the blindfolded participants), it would not be meaningful or of practical importance.

**Figure 3:**
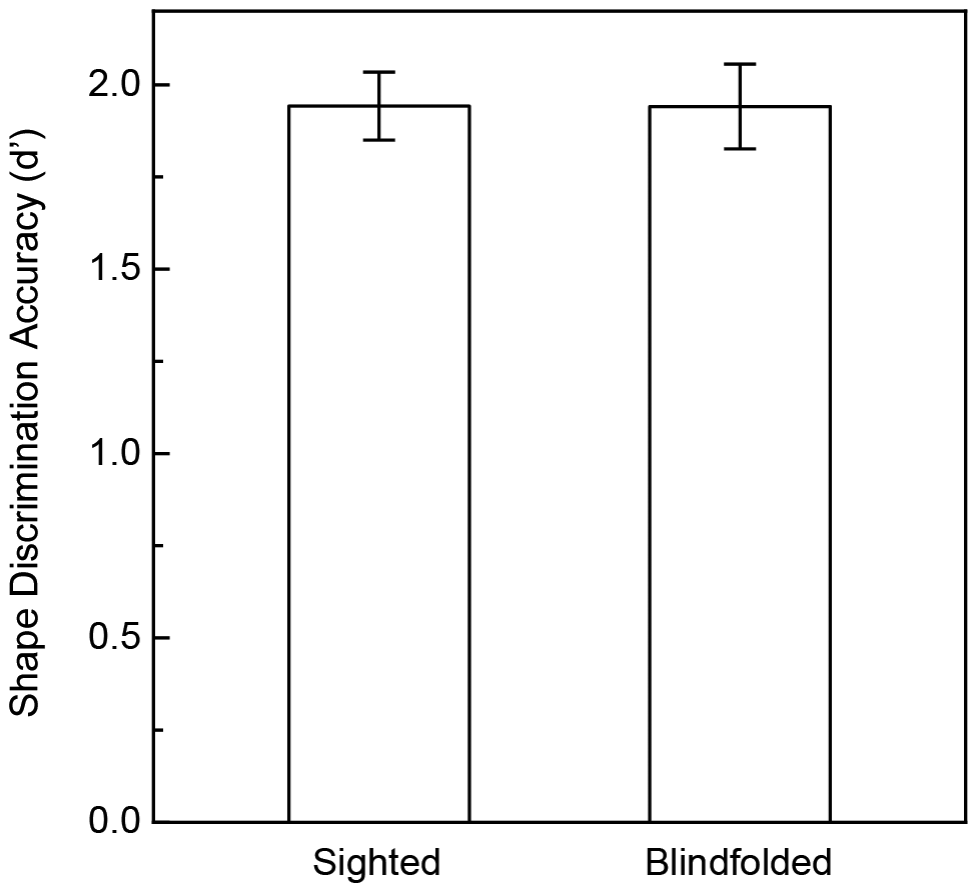
Experimental results. The participants' solid shape discrimination accuracies are plotted for both the sighted and blindfolded (i.e., visually deprived) participants. The error bars indicate ±1 SE.

The results for the solid shape haptic discrimination task are shown in Figure 3. While the blindfolded participants' shape discrimination accuracies were normally distributed (i.e., did not deviate from a normal distribution, W(14)  = 0.97, p>.05), the sighted participants' shape discrimination accuracies were not normally distributed (W(14)  = 0.76, p<.05). The deviation of the sighted participants' shape discrimination accuracies from a normal distribution largely occurred because of a single outlier: one sighted participant obtained a d′ score of 0.924, which was 2.96 standard deviations lower than the mean of the other 13 sighted participants (2.021). One, of course, could potentially exclude the outlier. However, we prefer to include all of the participants' data. Even though it has been demonstrated that parametric t-tests are ordinarily quite robust to violations of their assumptions [43], we chose to employ a distribution-free nonparametric analog of the t-test, the Wilcoxon Rank-Sum Test [44], [45], to test for any potential difference between the sighted and blindfolded groups of participants. It is clear from a visual inspection of Figure 3 that there was no significant difference between the shape discrimination abilities of the visually-deprived and sighted participants, and this was verified by the Wilcoxon Rank-Sum Test (W_x_  = 200, p = .91, 2-tailed). A power analysis [42] was conducted upon the results (difference in d′ scores) depicted in Figure 3. The analysis revealed that (in the best case) we would have needed a total sample of almost three million participants (2,835, 654) in order to have a 90 percent chance of detecting a difference this small (the average d′ scores for haptic shape discrimination in our experiment were 1.942 and 1.941 for the sighted and visually-deprived participants, respectively).

## Discussion

Prior research by four different laboratories, including our own [13]–[16], has demonstrated that blindness is associated with increased tactile acuity. It seems clear, therefore, that long-term visual deprivation can produce significant enhancements in tactile acuity. Fifty years ago, Zubek et al. ([23], p. 1592) reported that a week of visual deprivation produced “a pronounced increase in tactual acuity of the palm”; in particular, the 2-point thresholds of their participants declined by about 24 percent over the course of seven days. Recent investigators [46], [47] have concluded, however, that the measurement of 2-point thresholds does not constitute a valid measurement of tactile acuity. Therefore, we do not necessarily know at the present time whether a week of visual deprivation truly produces enhancements in tactile acuity (five days, however, of visual deprivation is sufficient to improve performance on a tactile Braille character recognition task [24], [25]).

In 2008, Leon-Sarmiento et al. [28] reported that the grating orientation discrimination thresholds of their “neurologically normal subjects” decreased by 23 percent following 45 minutes of visual deprivation. It is very important to note, however, that these investigators did not compare the performance of their visually deprived participants with similar control participants who were *not* visually deprived. It is thus likely that their participants' tactile acuity improved during the second measurement session as a result of practice (just as our participants' tactile acuity improved through practice and increasing experience with the task, see the current Figure 2) and not as a result of the 45 minutes of deprivation. Facchini and Aglioti [27] did compare the tactile acuity of visually deprived participants with a group of “non-deprived” participants; these investigators found a decrease in grating orientation discrimination thresholds from the first to second measurement session for their blindfolded participants, but not for the “non-deprived” control participants. The report of a more recent study [29], however, noted that Facchini and Aglioti visually deprived their “non-deprived” participants whenever their tactile acuity was being assessed – these authors (Wong et al.) then argued that the performance of Facchini and Aglioti's “non-deprived” participants was not necessarily representative, because these participants had not been tested under sighted (i.e., non-deprived) conditions. One puzzling aspect of the data obtained by Facchini and Aglioti (see their Figure 2) is that the tactile acuity of their non-deprived participants at the baseline initial measurement was already nearly as good as that (very similar, since the two error bars overlap substantially) exhibited by the deprived participants after 90 minutes of blindfolding. If 90 minutes of total light deprivation was needed for the deprived participants' tactile acuity to improve to a particular level, how could the non-deprived participants perform at a statistically similar level with no light deprivation? The results shown in Figure 2 of Facchini and Aglioti certainly indicate that 90 minutes of visual deprivation is not needed to obtain superior tactile acuity (since the “non-deprived” participants at baseline performed similarly to the deprived participants after 90 minutes of blindfolding).

The results of the current study were very different from those of Facchini and Aglioti [27]; in our experiment, the grating orientation discrimination thresholds became significantly lower during the second measurement session for both the blindfolded and sighted participants (see the current Figure 2). Our current results are, however, entirely consistent with those of Wong et al. [29]. Both our study and that of Wong et al. found that short-term visual deprivation (e.g., 90 minutes) does not lead to significant improvements in tactile acuity over and above the improvement exhibited by non-deprived control participants. Our investigation extended the study of Wong et al. by additionally evaluating the potential effects of short-term visual deprivation upon haptic solid shape discrimination. No previous psychophysical study has investigated deprivation and solid shape discrimination. As Figure 3 clearly indicates, there was no effect – blindfolded and sighted participants possessed equal solid shape discriminability.

As reviewed earlier, Weisser et al. [26] found that two hours of visual deprivation was sufficient to produce significantly higher performance (than that obtained for non-deprived control participants) for a tactile task involving the discrimination of 2-D shape (discriminating between upside-down letters “T” and “V”). The most robust difference in task-related cortical activity between their deprived and control participants occurred in visual extrastriate areas V3A and vIPS. Neurons within V3A are responsive to sharp edges or discontinuities in depth [48] (for their stimuli, Tsao et al. [48] used random-dot stereograms that depicted “checkerboard” patterns; prominent discontinuities in depth occurred at the linear boundaries between pairs of checks). In this context, it is probably important to note that the “V” and “T” stimuli used by Weisser et al. had polygonal shapes with distinct depth edges (there was a discontinuity in depth, 3.5 mm, between the V and T shapes and their background). The 3-D (solid) shapes that were used in the current experiment, bell peppers, were quite different – the surfaces of these objects are smoothly curved and continuous everywhere, even at the outer boundaries (i.e., the orientations of local surface regions change smoothly at the outer boundaries). It is possible that our smoothly-curved 3-D objects (lacking sharp discontinuities in depth at the outer boundaries) do not alter neuronal activity in V3A in the same way as the 2-D stimuli used by Weisser et al. (thus leading to differences in behavioral performance). Another obvious difference between the current study and that of Weisser et al. is that in the current experiment, our participants actively manipulated the bell pepper stimuli in order to make their judgments (a haptic judgment), whereas the tactile stimuli in the Weisser et al. study were manually pressed into the participants' immobilized right index fingerpad (passive tactile judgments). It is likely that active haptic manipulation and passive tactile judgments differentially activate many cortical regions, potentially leading to differential performance. In any event, it seems clear that short periods of visual deprivation can heighten the tactile discrimination of 2-D shape. Brief periods of visual deprivation also appear to produce enhancements for a number of auditory tasks, including the auditory ability to estimate distances to sound sources [49] and sound localization [50]. The results of the current study and that of Wong et al. [29], however, indicate that tactile grating orientation discrimination and haptic solid shape discrimination tasks are different, and are not appreciably affected by short periods of visual deprivation.

## Supporting Information

Dataset S1 **Individual participant results for the blindfold effectiveness test.**
(XLS)

Dataset S2 **Individual participant tactile acuities.** Each participant's tactile acuity was assessed using a grating orientation discrimination task.
(XLS)

Dataset S3 **Individual participant shape discrimination performance.**
(XLS)
